# The *Caenorhabditis elegans *D2-like dopamine receptor DOP-2 physically interacts with GPA-14, a Gα_i _subunit

**DOI:** 10.1186/1750-2187-7-3

**Published:** 2012-01-26

**Authors:** Pratima Pandey, Singh Harbinder

**Affiliations:** 1 Department of Biological Sciences, Delaware State University, Dover, DE 19901, USA

**Keywords:** Dopamine receptor, G-protein, GPCR, G*α*, split-ubiquitin, yeast two-hybrid, *Caenorhabditis elegans*

## Abstract

Dopaminergic inputs are sensed on the cell surface by the seven-transmembrane dopamine receptors that belong to a superfamily of G-protein-coupled receptors (GPCRs). Dopamine receptors are classified as D1-like or D2-like receptors based on their homology and pharmacological profiles. In addition to well established G-protein coupled mechanism of dopamine receptors in mammalian system they can also interact with other signaling pathways. In *C. elegans *four dopamine receptors (*dop-1, dop-2, dop-3 *and *dop-4*) have been reported and they have been implicated in a wide array of behavioral and physiological processes. We performed this study to assign the signaling pathway for DOP-2, a D2-like dopamine receptor using a split-ubiquitin based yeast two-hybrid screening of a *C. elegans *cDNA library with a novel *dop-2 *variant (DOP-2XL) as bait. Our yeast two-hybrid screening resulted in identification of *gpa-14*, as one of the positively interacting partners. *gpa-14 *is a G*α *coding sequence and shows expression overlap with *dop-2 *in *C. elegans *ADE deirid neurons. *In-vitro *pull down assays demonstrated physical coupling between dopamine receptor DOP-2XL and GPA-14. Further, we sought to determine the DOP-2 region necessary for GPA-14 coupling. We generated truncated DOP-2XL constructs and performed pair-wise yeast two-hybrid assay with GPA-14 followed by *in-vitro *interaction studies and here we report that the third intracellular loop is the key domain responsible for DOP-2 and GPA-14 coupling. Our results show that the extra-long *C. elegans *D2-like receptor is coupled to *gpa-14 *that has no mammalian homolog but shows close similarity to inhibitory G-proteins. Supplementing earlier investigations, our results demonstrate the importance of an invertebrate D2-like receptor's third intracellular loop in its G-protein interaction.

## Background

The vertebrate dopaminergic system controls a wide variety of physiological and neural functions such as locomotor activity, cognition, emotion, positive reinforcement, food intake, and endocrinal regulation. Aberrations in the levels of dopamine are associated with various disorders such as Parkinson's disease, schizophrenia, hypertension and psychosis [1,2]. In the recent past, efforts have been made to understand the role of dopaminergic signaling in invertebrate behavior and other biological activities due to their tractable genetics, evolutionarily conserved pathways and simpler nervous system. In *C. elegans *there are eight dopaminergic neurons in the hermaphrodite and an additional set of six neurons located in the tail of the male [3]. Dopamine has been found to participate in a wide array of nematode behaviors such as locomotion, food sensation, egg laying, defecation, and learning [4-8]. Dopamine receptors belong to a super-family of seven-transmembrane G-protein-coupled receptors (GPCRs) which are characterized by three intracellular loops (il_1-3_), three extracellular loops (ol_1-3_), an extracellular amino-terminus (N_t_) and cytoplasmic carboxy-terminus (C_t_) tail [9]. Dopamine receptors transduce external stimuli to the inside of the cell through their interaction with heterotrimeric G-proteins consisting of a guanosine nucleotide binding α-subunit, plus β and γ subunits. The G-protein subunits can further interact with downstream proteins to produce specific effector response(s). An impetus to the clinical advancement came from the development of agonists and antagonists of dopamine receptors as new drug targets [10].

In the fully sequenced *C. elegans *genome, four dopamine receptors have been identified. Based on their sequence profiles and pharmacological properties DOP-1 is classified as a D1-like receptor, DOP-2 and DOP-3 are D2-like receptors and DOP-4 is an invertebrate-specific receptor [11,12]. D1-like receptors transduce signals through G*α*s-subunits to increase adenylate cyclase activity whereas D2-like receptors function through G*α*i/o coupling which results in the inhibition of adenylate cyclase [13,14]. The *C. elegans *genome encodes for 21 G*α*, 2 G*β *and 2 G*γ *proteins. Four of the G*α *genes *goa-1, gsa-1, egl-30, gpa-12 *are similar to mammalian classes of G*α *family G*α*i/o, G*s*, G*q *and G*12*, respectively. The remaining 17 G*α *do not display close similarity to any mammalian class but are somewhat related to G*α*i/o proteins [15]. The mammalian D2 receptor, DRD2 has 3 isoforms, termed D2Short, D2Long and D2Longer [16,17] that arise from alternative splicing. Studies done in the past suggest that the third cytoplasmic or intracellular loop (il_3_) of DRD2 plays a crucial role in determining the coupling specificity of receptor/G-protein interaction [18,19]. In *C. elegans, dop-2 *receptor was initially cloned and characterized by Suo *et. al*., 2003 [11]. They reported two isoforms CeDOP-2S and CeDOP-2L encoded by *dop-2a *and *dop-2b *with differences in the size of the third intracellular loop akin to observations in mammalian DRD2. Pharmacological characterization of CeDOP-2S and CeDOP-2L showed that they have high binding affinity for dopamine and can inhibit adenyl cyclase activity [11]. At present little is known about the molecular basis of signaling pathways activated through *dop-2*, and other dopamine receptors in *C. elegans*. Mammalian studies suggest higher complexity in dopamine receptors as they can couple to other signaling pathways. In addition to their effect on adenylate cyclase activity dopamine receptors modulate calcium concentrations, potassium channel activity and release of arachidonic acid (10). Investigating dopamine receptors in model organism such as *C. elegans *with powerful genetics and a tractable, compact nervous system can provide new insight into molecular mechanisms of dopaminergic signaling as well as allow for follow-up genetic and behavioral experiments.

In this study, we identified a specific G*α *interacting partner for the DOP-2 receptor by utilizing split-ubiquitin based yeast two-hybrid system. In the process we found a third (and longest) splice variant of *dop-2 *referred as *dop-2c *(KO9G1.4c) that codes for CeDOP-2XL (extra-long variant). We used this extra-long variant for screening a *C. elegans *cDNA library which resulted in the identification of GPA-14 as one of the DOP-2 interacting protein. GPA-14 belongs to a group of invertebrate G*α *proteins with no mammalian homologs but are most close to Go/i class. The interaction between CeDOP-2XL and GPA-14 was confirmed by pair-wise two-hybrid assays and direct *in-vitro *protein-protein interactions. Our domain interaction results suggest that both il_2 _and il_3 _domains of CeDOP-2XL are involved in GPA-14 coupling whereas il_3 _domain is critical for this interaction.

## Materials and Methods

### DNA constructs for yeast two-hybrid

Total RNA was isolated from *C. elegans *(wild-type Bristol N2) using a column based protocol and reverse transcription was performed using a commercial kit (Roche, Indianapolis, IN). *dop-2c *(KO9G1.4c) coding region was amplified using the primers (detailed in Additional file 1) and cloned in pBT3-SUC and pBT3-STE vectors (Dual Systems Schlieren, Switzerland) to produce a fusion protein with C-terminal part of ubiquitin (Cub) followed by LexA-VP16 transcription factor. *C. elegans *cDNA library (Dualsystems) consisted of adult *C. elegans *cDNA cloned in prey vector pPR3-N as a fusion protein with N-terminal part of ubiquitin (NubG: mutated N-terminal half of ubiquitin). Interaction between the fusion proteins reconstitutes the split ubiquitin leading to the cleavage and release of transcription factor LexA-VP16 by ubiquitin-specific proteases and results in LexA-VP16 activated expression of lacZ, HIS3, and ADE reporter genes. *gpa-14 *amplified fragments were cloned into prey vectors pPR3-N and pPR3-STE. DOP-2 truncated constructs DOP-2IL-CI (amino acids 64-849), DOP-2IL-CII (124-849), DOP-2IL-CIII (224-849), DOP-2IL-CIV (821-849) were amplified and cloned into bait vector pBT3-SUC. pBT3-SUC carries a signal sequence derived from the *Saccharomyces cerevisiae *invertase-*SUC2 *gene that ensures proper insertion of bait into yeast membranes. Constructs were verified by DNA sequencing. Sequences of oligonucleotides used in this study are provided in Additional file 1.

### Yeast two-hybrid screening

Yeast transformations and library screenings were done according to the Dualsystems protocols. In our assays, pAI-Alg5 was used as positive control. It expresses the full-length resident ER protein, Alg5 fused to the NubI portion (the N-terminal 38 amino acids of wild type ubiquitin). Due to the strong affinity of NubI for Cub, any bait that is co-expressed with pAI-Alg5 and that is correctly integrated into the membrane grows well on selective medium thus act as a positive control. Similarly, pDL2-Alg5 was used as negative control which expresses Alg5 fused to NubG, with no affinity for Cub, and Alg5 by itself does not interact with protein of interest in bait. Full-length *C. elegans dop-2c *cDNA cloned into bait vector pBT-3 STE was transformed by PEG/LiAc transformation method into *S. cerevisiae *strain NMY51 and transformants were selected on SD-Leu medium at 30°C. Yeast cells containing the bait were grown in the selective medium and transformed with *C. elegans *cDNA library. To optimize the screening stringency, selection plates were supplemented with increasing concentrations of 3-AT (0, 1, 2.5, 5, 7.5, and 10 mM). Finally transformants were selected on medium lacking adenine, histidine, leucine, and tryptophan (-Ade-His-Leu-Trp) + 1 mM 3-AT selection plates. Putative interactions were verified by X-gal filter lift-off assay. Prey plasmids were rescued from positive interacting clones using zymoprep plasmid isolation kit and transformed into *E.coli *(DH5*α*). The bacterial DNA was used for sequence analysis.

### GST tagged pull-down assay

Full-length *gpa-14 *was PCR amplified and cloned downstream to the GST tag in a pET-24a based vector. *dop-2c *including the 3' polyA (30 nucleotides) was amplified and cloned into pIVEX2.3d between the *Nco*I and *Sac*I sites. *dop-2c *was expressed *in-vitro *by using a coupled transcription/translation kit (Promega, Madison, WI, USA). GST:GPA-14 fusion protein was purified using Magne-GST particles following the manufacturer's instructions (Promega). Protease inhibitor cocktail mix (Thermo-Fisher, Waltham, MA) was added to the binding/wash buffer and elution buffer. Interaction was tested by adding 5 μl of eluted GST:GPA-14 fusion protein to 20 μl of *in-vitro *translated DOP-2 in the binding buffer containing 0.2% BSA and incubated for 6 hrs at 4°C. The resin was then washed three times with binding buffer and the interacting complex was resolved by SDS PAGE and detected colorimetrically by translation detection system that detects biotinylated lysyl-tRNA incorporated into DOP-2 during translation (Promega).

### Generation of histidine-tagged Gα-proteins and truncated dopamine receptor constructs

For constructing histidine-tagged fusion proteins first a His-tag was cloned into pTNT vector (Promega) to obtain pTNT-HIS vector. Full-length cDNAs were amplified for *gpa-14, gpa-15 *and *gsa-1*, and cloned in pTNT-HIS. To generate truncated DOP-2 constructs, [DOP-2-CV, consisting of il_1_+il_2 _(1-182 amino acids), and DOP-2-CVI which contained the il_3_+C_t _region (183-849 amino acids)] their respective cDNA regions were amplified and directionally cloned into pTNT vector. Oligos used for amplification are mentioned in Additional file 1. The above constructs were used for *in-vitro *translation. Binding reaction was performed by mixing *in-vitro *translated histidine-tagged GPA-14 (or GPA-15/GSA-1) and DOP-2 truncated constructs. The proteins were then allowed to interact at 4°C with agitation in 300 μl of binding buffer (20 mM sodium phosphate, 500 mM NaCl, 5% BSA) and protease inhibitor cocktail mix. After 2 h, 60 μl of nickel resin (MagZ particles, Promega) was added, and the reaction mixture was further incubated for 2 h at 4°C. The resin was washed three times with binding buffer and twice with binding buffer minus BSA [20]. The bound proteins were eluted with 20 μl of sample buffer and diluted 1:10 and heated at 70°C for 10 min. Samples were resolved by SDS-PAGE (6.5% gel), and interacting proteins were detected colorimetrically.

## Results

### Yeast two-hybrid library screening identified putative Gα_i _protein GPA-14 as a DOP-2 interacting protein

**Figure 1:**
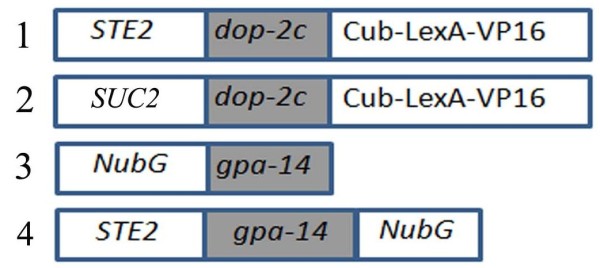
**Constructs used for split ubiquitin assays**. Bait protein fusion was made with the C-terminal part of ubiquitin (Cub) followed by artificial transcription factor LexA-VP16 at the C-terminus of DOP-2XL (1, 2). Prey protein fusions were constructed with the mutant N-terminal part of ubiquitin (NubG) either at the C terminus of GPA-14 (3) or at the N-terminus (4). If bait and prey interact NubG and Cub are forced into close proximity, resulting in the reconstitution of split-ubiquitin and release of LexA-VP16 transcriptional factor that leads to transcriptional readout, resulting in growth of yeast on selective medium and color development in a β-galactosidase assay.

In order to identify the proteins that interact with the membrane receptor DOP-2, we used the split-ubiquitin based yeast two-hybrid system designed to investigate the interactions of membrane proteins. Our analysis of the DOP-2 amino-acid residues for locating the N-terminal cleavable sequence using SignalIP 3.0 bioinformatics tool [21] predicted that DOP-2 does not have any cleavable sequence. Thus, it was decided to amplify full-length *dop-2 *and clone it into pBT-3 STE vector (STE2 sequence improves translation of bait). In the process of reverse-transcriptase PCR amplification of *dop-2 *full-length cDNA we serendipitously amplified an additional splice variant of *dop-2 *(KO9G1.4c), besides the 2 known variants *dop-2a *and *dop-2b *[11]. This third transcript *dop-2*c has 27 additional nucleotides at the 3' end of exon-8 neighboring the exon:intron junction. To differentiate from the 2 known variants we named the *dop-2*c protein product as CeDOP-2XL. We used this novel CeDOP-2XL as bait to screen an adult *C. elegans *cDNA library. *dop-2c *ORF was fused to Cub in pBT-3 STE vector (Figure 1) and used for screening *C. elegans *cDNA library with cDNA fused with mutated form of N-terminal ubiquitin (Nub G). Selection of positive interacting partners was done on plates lacking ade, his, leu and trp and further also tested by *lacZ *expression. This selection process yielded 450 positive clones. Follow up with sequencing based screening of the CeDOP-2XL interacting clones lead us to the open-reading-frame B0207.3 that was the most abundantly represented sequence amongst the sequenced clones (~20%; data not shown). This sequence is interesting as it is predicted to code for a G*α*-subunit, GPA-14, and akin to other 7-transmembrane receptors DOP-2 is likely to act through a G-protein coupled pathway. The screen did not yield any other G-protein subunits, and all the different *gpa-14 *clones obtained were either full length, or deemed full length; the latter contained the N-terminal and the middle portion of *gpa-14 *coding sequence. There are previous reports that G*α *regions responsible for interaction with receptors are located at the N-terminus, C-terminus or at the central region of the protein [22,23].

**Figure 2:**
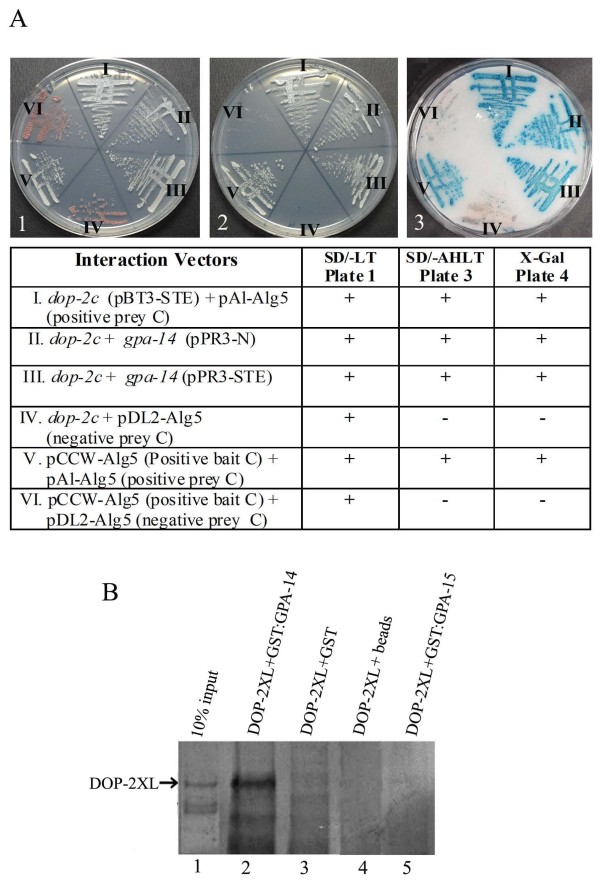
**Interaction studies of dopamine receptor DOP-2XL with Gα protein GPA-14**. (A) Split-ubiquitin based pair-wise interaction. Yeast two-hybrid screen with full-length DOP-2XL yielded GPA-14 as a positive interaction partner that was validated by pair-wise interaction between *dop-2c *(cloned in pBT3-STE) and *gpa-14 *(cloned in pPR3-N and pPR3-STE). The interaction was determined by growth assay on selective growth media and X-gal filter overlay assay. For the growth assay, selected yeast colonies were streaked onto a master plates (plate 1, SD/-Leu-Trp), and selective plates (plate 2, SD/-Ade-His-Leu-Trp) containing 1 mM 3-AT. Absence of adenine from the selection medium is useful since in the absence of a protein-protein interaction, the *ADE2 *reporter gene is not transcribed and therefore, a red-colored intermediate accumulates in the adenine metabolic pathway and gives red color to the colony (plate 1). To perform X-gal filter lift-off assay (plate 3, X-gal) colonies from master plate were replica transferred to 3 mM filter paper and assay was performed as described by Dualsystems. The growth results and X-gal assay results are interpreted as good interaction (+), weak interaction (+/-), or no interaction (-). (B) *In-vitro *interaction between DOP-2XL and GPA-14. GST-tagged GPA-14 and GPA-15 (~ 72 KDa) was purified by immobilization with paramagnetic GST particles (data not shown). DOP-XL protein (~94 KDa) expression was obtained by *in-vitro *transcription/translation method (lane1). DOP-2 XL was incubated either with purified GST:GPA-14 or with purified GST:GPA-15, GST, paramagnetic beads, as controls. At the end of the incubation the beads were collected from the mixtures and washed. The eluted samples were applied to SDS PAGE and transferred to PVDF membrane and probed colorimetrically. *In-vitro *synthesized DOP-2XL was pulled down with Magne-GST beads when incubated with GST:GPA-14 (lane 2) but not when incubated with GST alone (lane 3), or with magnetic beads only (lane 4); however, a weak band is visible with GST:GPA-15 (lane 5) which is likely due to non-specific interaction.

To further test the DOP-2XL and GPA-14 interaction we performed pair-wise interaction assays. The full-length constructs for the latter were generated by directly amplifying *gpa-14 *cDNA and cloning in pPR3-N and pPR3-STE vectors to obtain distinct C-terminal fusions (Figure 1). These fusions were separately transformed into yeast strain harboring *dop-2c *bait construct, and the inference of interactions revealed that *dop-2c *and *gpa-14 *gene products exhibit positive interaction as shown by growth assays on minimal media (Figure 2A plate 2, sectors II, III) and X-gal filter assay (plate 3, sectors II, III). This interaction is specific since *dop-2c *failed to bind the yeast ER protein Alg5 in control prey pDl2-Alg5 (Figure 2A plate 2, sector IV). Results from two-hybrid screening and pair-wise interaction assays demonstrate that GPA-14 is an interacting partner for DOP-2 in yeast.

### Direct protein-protein interaction between DOP-2XL and GPA-14

To confirm that DOP-2XL binds to GPA-14, the latter was tagged with GST at its N-terminus. The GST:GPA-14 fusion protein was expressed in *E. coli *and following lysis, the protein fractions were resolved by SDS-PAGE (data not shown). We obtained GST tagged GPA-14 as a partially soluble protein that was immobilized to magnetic GST beads and used as a bait to pull down DOP-2XL. DOP-2XL was expressed as prey by *in-vitro *coupled transcription/translation system. Translated DOP-2XL was allowed to interact with GST:GPA-14 and control proteins at 4°C. The post interaction complex was successfully pulled down using glutathione immobilized paramagnetic particles and the eluted samples were resolved by SDS-PAGE. Colorimetric detection of the bound complex showed that the paramagnetic particles could pull down DOP-2XL only in the presence of GST-tagged GPA-14 (Figure 2B, lane 2). However, when DOP-2XL was allowed to interact with GST or incubated alone with paramagnetic beads no DOP-2XL was detected in pulled down fractions (Figure 2B, lanes 3, 4-check lanes). A very faint DOP-2XL like band is observed in the lane with GST-tagged GPA-15, which may possibly be due to cross-reactivity of conserved domains present in GPA-15 and GPA-14. While *gpa-15 *is not known to co-express with *dop-2*, it does share a partial expression overlap with *gpa-14 *in the PHA and PHB sensory neurons, indicating that they may compete for interacting with other receptors [15]. In summary, the above results confirm the *in-vitro *physical interaction between DOP-2XL and GPA-14.

### The third Intracellular domain of DOP-2XL is essential for GPA-14 binding

**Figure 3:**
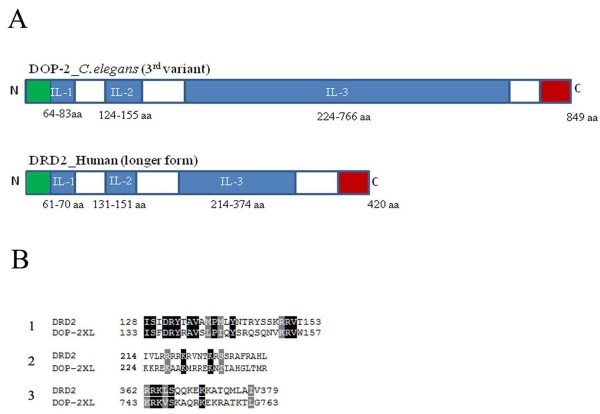
**Domain analysis of human DRD2Longer and *C. elegans *DOP-2XL dopamine receptors**. (A) Schematic representation of receptor domains. DRD2Longer and CeDOP-2XL intracellular loop (il) are represented as blue shaded boxes and N_t _and C_t _are shown as green and red boxes, respectively. The numbers indicate the position of each intracellular loop in both the receptors. DOP-2XL il_3 _(224-766 aa) consists of 546 amino acid residues that is more than 3 times the length of DRD2 il_3 _(214-374 aa) with 160 amino-acid length. il_1_and il_2 _were almost comparable in size with less than 10 amino-acid difference. (B) Sequence comparison of receptor domains. Sequence alignment of DOP-2XL (WormBase accession no WP:CE45633) and DRD2Longer (UniProt accession no P14416-3) was performed based on their predicted GPCR structures. The domains used for alignment were il_2 _and il_3 _(il_3_N) & (il_3_C) regions found to be necessary for Gi coupling.

It is evident from the above results that DOP-2XL interacts with GPA-14, and the interaction observed in our indirect two-hybrid system is in agreement with the direct *in-vitro *protein-protein interaction. Previous efforts to define GPCR interacting domains have used

chimeric constructs and small fragments of GPCRs [24-26]. In order to undertake domain interaction studies of DOP-2, initially we performed sequence analysis using CeDOP-2XL as a query. BLAST [27] analyses showed 33% sequence similarity with the well studied human D2 receptor DRD2. Domain structure for CeDOP-2XL was identified by PlantsP software (http://plantsp.genomics.purdue.edu) and in case of DRD2 Uni ProtKB/SWISS-Prot database information was used (Figure 3A). We performed a search for conserved structural motifs within the suggested structure of the CeDOP-2XL and found that the additional 9 amino-acid residues (GDLPLPMLL) in CeDOP-2XL variant formed part of the large intracellular loop il_3 _and the resulting motif does not display similarity to any known motif based on Pfam analysis [28]. The DOP-2 il_3 _(546 amino-acid residues) is more than 3 times the length of DRD2il_3 _(160 amino-acid residues), whereas il_1 _and il_2 _are comparable in length with less than 10 amino-acid residue difference (Figure 3A). Previous studies, where synthetic peptides were used for receptor-G-protein interactions [19,25], have suggested that il_2 _and N and C terminal regions in il_3 _encompasses the major domains necessary for coupling to inhibitory G-protein whereas the il_3 _central region is important for the stimulatory branch [29]. Thus, the corresponding regions of CeDOP-2XL were selected within the intracellular loops for BLAST analysis. We found that in il_2 _and il_3 _there was high level of conservation and the amino-acid sequences suggested to be necessary for G-protein interactions were conserved (Figure 3B). The remaining region in il_3 _exhibited only minimal similarity to its mammalian homolog at the amino-acid level.

**Figure 4:**
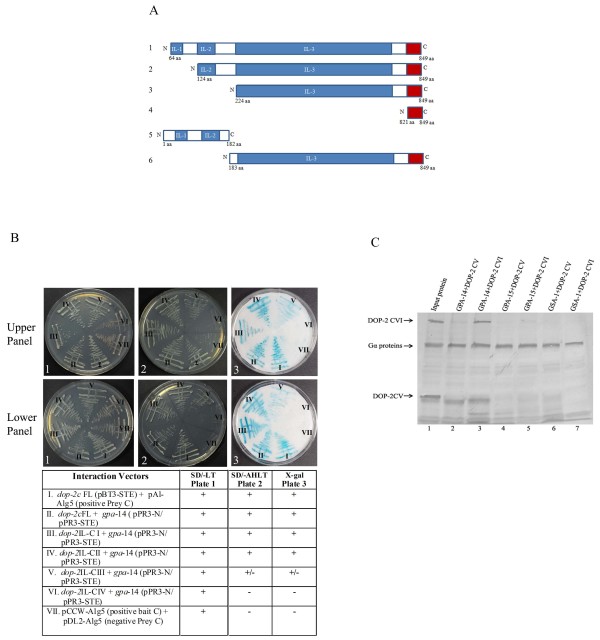
**DOP-2 third intracellular loop (il_3_) interacts with GPA-14**. (A) Schematic representation of DOP-2XL truncated protein constructs. DOP-2XL truncated region in each construct is indicated by amino-acid residue positions. Panel 1-4 are constructs, DOP-2IL-CI- DOP-2IL-CIV for yeast two-hybrid interactions. DOP-2IL-CI (amino acids 64-849) consisted of all the domains but N-terminal domain and transmembrane 1 was absent. DOP-2IL-CII protein (124-849 amino acids) starts at the junction of predicted transmembrane 2 and il2. DOP-2IL-CIII (amino acids 224-849), truncated protein was lacking domains il1 and il2. DOP-2IL-CIV truncated protein (821-849 amino acids) is just the C-terminal intracellular region beyond transmembrane 7 without any loop. Panels 5 & 6 represent DOP-2-CV and DOP-2-CVI used for His pull down assay. DOP-2-CV (amino acids1-182) expressed il_1_+il_2 _and DOP-2-CVI (amino acids 183-849) consisted of il_3_+C_t _region (B) Interaction between truncated DOP-2 bait constructs and GPA-14 prey proteins. *dop-2c *FL was cloned in pBT3-STE and truncated domain constructs were made in pBT3-SUC vector to be used as bait, *gpa-14 *was cloned in two different prey vectors pPR3-N (upper panel) and pPR3-STE (lower panel). Interaction was determined by growth assay on selective growth media, plate 1 (SD/-Leu-Trp), plate 2 (SD/-Ade-His-Leu-Trp), X-Gal filter overlay assay, plate 3 (X-gal) and demonstrated as (+) good interaction; weak interaction (+/-); no interaction (-). Sector-I represents positive interaction control, sector-VIII was negative-control in both the cases, and sectors II, III, IV, V and VI were test interactions. (C). Interaction between DOP-2 truncated constructs and G*α*-proteins by His pull-down assay. Lane 1 shows *in-vitro *translated G*α*-His fusion proteins (GPA-14, GPA-15 and GSA-1) and DOP-2XL constructs (CV, CVI). DOP-2XL constructs were incubated with His tagged GPA-14 or with control proteins His:GPA-15/His:GSA-1 at 4°C. After allowing these proteins to interact, nickel resin (Mag Z particles, Promega) was added. At the end of this incubation, beads were collected by using magnetic stand and washed and eluted, and the sample was resolved by SDS-PAGE. Colorimetric detection of interaction complex showed that DOP-2-CVI (lane 3) but not DOP-2-CV (lane 2) was pulled down with GPA-14-His protein. Both DOP-2-CV & DOP-2-CVI showed no binding with control G*α *proteins GPA-15 and GSA-1 (lanes 4, 5, 6 & 7).

To ascertain the specific DOP-2XL intracellular loops responsible for these interactions, we prepared four *dop-2c *constructs with sequential deletions (Figure 4A, panels 1, 2, 3, 4) and tested for their interaction with GPA-14. These intracellular domain constructs were co-transformed with full-length *gpa-14 *and interactions were determined by growth assays on minimal media (Figure 4B, plate-2) and X-gal filter assays (Figure 4B, plate-3). These data show that the deletion of N-terminal region and il_1 _did not affect the interaction of the DOP-2XL with GPA-14 (Figure 4B, plate-2, sectors III, IV). When il_2 _was removed there was a weak interaction (Figure 4B, plate-2, sector-V); however when il_3 _was truncated the interaction between the proteins was lost (Figure 4B, plate-2, sector-VI).

Since our data from the yeast two-hybrid assays indicated that il_2 _and il_3 _are critical for GPA-14 binding, we further confirmed these interactions by pull-down assays. GPA-14 and other control G*α*-proteins were expressed as fusion proteins with His-tags at their N-termini. Two constructs containing distinct DOP-2XL domains were generated in pTNT vector (Figure 4A, panels 5, 6). The DOP-2XL truncated constructs and His-G*α *fusion constructs were translated *in-vitro *in the presence of biotinylated lysyl-tRNA. We tested the ability of constructs DOP-2-CV and DOP-2-CVI to bind with His-tagged GPA-14 fusion proteins for pull-down assays using paramagnetic histidine-beads. Separate negative control experiments were performed using two other Gα-proteins from *C. elegans*, namely GSA-1 and GPA-15, which did not show up in our two-hybrid screen. Compared to GPA-14, both these proteins show an overall sequence similarity of ~44% and sequence identity of ~25% at the amino acid level. DOP-2-CVI (il_3_+Ct region) interacted specifically with GPA-14 (Figure 4C, lane 3) and not with GPA-15 or GSA-1 (Figure 4C, lanes 5, 7). However DOP-2-CV (il_1_+il_2_) could bind neither to GPA-14 nor to the control proteins GSA-1 and GPA-15 (Figure 4C, lanes 2, 4, 6). Hence we determined that the DOP-2XL receptor region primarily responsible for interacting with GPA-14 lies in il_3_. This is based on the observation that only DOP-2-CVI (il_3_+Ct) but not DOP-2-CV (il_2_+il_3_) were recognized by GPA-14.

## Discussion

In the past there have been significant efforts to understand the complex dopamine receptor pathways in vertebrates. With advancement in understanding dopaminergic signaling it became evident that dopamine receptors are complex sensory proteins that not only activate G-proteins but also interact directly or indirectly with components of various other pathways. In that regard, *C. elegans *can serve as a useful model system since the dopaminergic system of this organism shows remarkable conservation with the mammalian system and other invertebrates. The focus of this study was to identify specific interacting partners for DOP-2 using this D2-like receptor protein as bait in a yeast-two hybrid system. The DOP-2 receptor being a membrane-bound structure, it was important to use an assay system that could identify protein interactions at the membrane. We utilized a split-ubiquitin based yeast two-hybrid system with vectors optimized for membrane targeting using SUC2 cleavable sequences and improved bait translation with STE2 sequences [30]. In the process of *dop-2 *cDNA cloning we identified the extra-long version of the *C. elegans *DOP-2 receptor. This CeDOP-2XL may represent the invertebrate equivalent to the longest isoform of the human DRD2 receptor. Use of the CeDOP-2XL as a bait in the above system allowed us to identify GPA-14 as a potential interacting partner for DOP-2. Interestingly, *dop-2 *and *gpa-14 *display overlapping expression in the ADE pair of dopaminergic neurons [8,15]. It was intuitively logical to perform further studies with GPA-14 as it is one of the 17 *C. elegans *proteins with sequence similarity to the Gi/o family, and D2-like receptors in mammals are known to inhibit adenylyl cyclase activity by coupling with G*α*i [14]. We authenticated our screening result through pair-wise interactions between DOP-2XL and GPA-14 (Figure 2A). In the past, efforts have been made to identify the interacting partners for G-proteins in *C. elegans *by using different G-proteins as bait in classic yeast two-hybrid screens. These efforts identified interacting partners for 4 G*α*-proteins but were unable to identify interacting partners for GPA-14 and the remaining G-proteins [31]. However, our strategy might have given us an edge since we used the split-ubiquitin system that is specifically designed for membrane proteins, and G*α*i/o proteins are known to interact with membrane receptors. Secondly, we used an adult *C. elegans *library which is likely to have a better representation of proteins that interact with adult expressed DOP-2 receptor, as compared to mixed stage library used by earlier investigators [31]. Taken together, our studies provide support for the existence of a functional trimeric G-protein complex in *C. elegans*. Interactions between DOP-2XL and GPA-14 were also validated by *in-vitro *interactions using GST pull-down assay (Figure 2B). The interaction between DOP-2 and GPA-14 in the absence of activating ligand can be based on the phenomenon of pre-coupling, in which the GPCR and the heterotrimer form a stable complex even in the absence of a ligand [25,32]. Our results support the assertion that the *C. elegans *dopamine receptor DOP-2XL functions as a GPA-14 coupled receptor that may play a central role in conferring specificity to pathways activated through this receptor.

In humans, the three reported genomic variants of D2 receptor differ in their ligand binding specificity and exert differential effects on downstream effectors [29,33,34]. The G-protein coupling efficiency and pharmacological properties of CeDOP2S and CeDOP2L variants have been reported to be comparable to their respective human DRD2 variants [11]. These splice variants and the new variant reported in the current study differ primarily in their third intracellular loop, as seen in human DRD2 variants, supporting the conjecture that the third intracellular loop might play an important role in their distinct functions. To follow-up, we investigated the receptor domains necessary for interaction with GPA-14. Earlier studies have indicated that the entire structure of the dopamine receptor molecule is not necessary for the interaction with G-proteins and short peptides containing critical amino acid sequences from the receptor were found to be able to effectively interact with the Gα subunits [19,29,35]. Therefore, we made sequentially truncated constructs in which the 3 intracellular loops were successively deleted. Our results from growth assays and X-gal assays showed that deletion of il_1 _did not disrupt the interaction between the coupling partners whereas deletion of il_2 _had subtle effects and il_3 _deletion resulted in loss of interaction (Figure 4B). The C_t _cytoplasmic region did not show any interaction by itself with GPA-14 as reported for mammalian D2 receptors [26,28]. Extensive investigations of the catecholamine receptors using site-directed mutagenesis, receptor chimeras, peptides and antibodies, have implicated the third intracellular loop (il_3_), especially its amino and carboxyl-terminal regions as an important, but perhaps not exclusive determinant of selective G-protein coupling [19,28]. Encouraged by these results we analyzed the DOP-2XL sequence and observed that N-terminal and C-terminal part of il_3 _showed high sequence conservation whereas other il_3 _regions had little similarity to DRD2. Our analysis of DOP-2XL truncated intracellular loop constructs by *in-vitro *interactions as shown in Figure 4C, suggested that GPA-14 could not interact strong enough with il_1_+il_2 _loop construct (DOP2-CV) whereas il_3_+C_t _domains in DOP2-CV together could be pulled down by His:GPA-14 fusion protein. Therefore, our results present il_3 _as the essential loop for DOP-2 interaction with GPA-14.

While our yeast two-hybrid results indicate a weak interaction of il_2 _with GPA-14, the il_2 _domain did not show interaction in the pull-down experiments (Figure 4B, C). As mentioned above, the *in-vitro *interactions represent the pre-coupled state of the two proteins. Thus, in the wake of previous studies and our results, we suggest that it is likely that il_3 _by itself may not be sufficient for functional coupling that leads to cAMP inhibition and that the second intracellular domain may play a role in GPCR and G*α *coupled signaling *in-vivo *[18,19,28,35]. Future experiments focused on understanding the role of specific DOP-2 domains with respect to the effector responses of the D2 receptor will be valuable. Both dop-2 and gpa-14 are known to express in the ADE pair of dopaminergic neurons, and RIA interneurons. *dop-2 *loss-of-function mutants are deficit in associative learning [36], therefore based on our results we predict that *gpa-14 *loss-of-function mutants should display a similar phenotype. It will also be interesting to generate *dop-2 *mutants with the individual amino-acid substitutions in key il_3 _residues to analyze their effects in terms of interaction with GPA-14 as well as in terms of phenotypic effects on worm behavior.

## Conclusion

The complexity of dopamine receptor pathways have hindered a clear understanding of the molecular components involved in dopamine signaling. In the current investigation we have successfully demonstrated that in *C. elegans *the novel dopamine receptor DOP-2XL interacts with a putative G*α*i protein GPA-14. Our data further suggested that third intracellular loop of DOP-2XL is necessary for GPA-14 coupled interaction. Thus our study provides further evidence that DOP-2 belongs to D2-like family since it interacts with inhibitory Gα protein that is characteristic of this group of dopamine receptors. Our results have also opened avenues to decipher signaling pathways for other dopamine receptors and towards advancing *C. elegans *as a live *in-vivo *model system for diseases associated with dopaminergic signaling.

## Abbreviations used

GPCRs: G-protein coupled receptors; G**-**protein: Guanine nucleotide-binding protein; G*α*s: stimulatory G-alpha subunit; G*α*i: Inhibitory G-protein; il: intracellular-loop; ol: extracellular-loop; N_t_: extracellular N-terminal domain; C_t_: cytoplasmic C-terminal domain; DOP-2: *C. elegans *D2-like dopamine receptor; GPA-14: *C. elegans *G*α*i-subunit; 3-AT: 3-amino triazole; GST: glutathione-S-transferase

## Competing interests

Neither of the authors (PP and SH) have competing interests.

## Authors' contributions

Conceived project: SH. Designed experiments: PP and SH. Performed experiments: PP. Wrote manuscript: PP and SH. Both authors have read and approve the final manuscript.

## Authors' information

PP (Post-doctoral Associate) and SH (Associate Professor) affiliated with the Department of Biological Sciences, Delaware State University, Dover, DE 19901

## Supplementary Material

Additional file 1 **Sequences of oligonucleotides**. Oligonucleotide sequences used for PCR amplification of DNA fragments to generate constructs used in the study. Click here for file
