# Supplementary material for: The Caenorhabditis elegans D2-like dopamine receptor DOP-2 physically interacts with GPA-14, a Gαi subunit
Source: J Mol Signal. 2012 Jan 26;7:3. doi: 10.1186/1750-2187-7-3 (PMC3297496; doi:10.1186/1750-2187-7-3)
Supplement: Additional file 1 — Sequences of oligonucleotides. Oligonucleotide sequences used for PCR amplification of DNA fragments to generate constructs used in the study. [file 1750-2187-7-3-S1.DOCX]

**Additional File-1:** Sequences of oligonucleotides: Oligonucleotide sequences used for PCR amplification of DNA fragments to generate constructs used in the study.

| Constructs/ Primer name 5’🡪3’ Sequence |
| --- |

**Yeast two-hybrid bait constructs**

dop-2c FL/ pBT3SUC

PS13 F GGCCATTACGGCCAgtggaattcctttgaactac

PS14 R GGCCGAGGCGGCCCCgacatgcgcctgcttgttactg

dop-2c/ pBT3 STE

PS 21 F GGCCATTACGGCCTTgaggccggagagacatggaat

PS22 R GGCCGAGGCGGCCCCgacatgcgcctgcttgttact

**Yeast two-hybrid prey constructs**

gpa-14 FL /pPR3-N

PS17 F GGCCATTACGGCCAatggcgtttagttgttttgaca

PS18 R GGCCGAGGCGGCCttatgaaaggcccgttgcttttg

Gpa-14FL/pPR3-STE

PS19 F GGCCATTACGGCCTTgcgtttagttgttttgacaag

PS20 R GGCCGAGGCGGCCCCtgaaaggcccgttgcttttgc

**DOP-2 domain constructs**

**Dop-2-ILCI**

PS44F ATTAACAAGGCCATTACGGCCcggtaccgagcactg

**Dop-2-ILCII**

PS46F ATTAACAAGGCCATTACGGCCgcttctattcttctt

**Dop-2-ILCIII**

PS47F ATTAACAAGGCCATTACGGCCcgagaaaaggcagcta

**Dop-2-ILCIV**

PS48F ATTAACAAGGCCATTACGGCCaacacggaattccgt

PS45R AACTGATTGGCCGAGGCGGCCCCgacatgcgcctgctt

**His pull down constructs in pTNT**

**DOP-2-CV**

PS89 F (*Xho*I site) CCGCTCGAGatggaggccggagag

PS91 R (*Sma*I site) TCCCCCGGGttaggttagcatctggtg

**DOP-2-CVI**

PS92 F (*Xho*I site) CCGCTCGAGatgccaccagatgctaacc

PS90 R (*Sma*I) TCCCCCGGGttagacatgcgcctgc

**His:GSA-1**

PS93 F (*Xba*I) CTAGTCTAGAgggtgcgtcggcgctggcgc

PS94 R (*Sma*1 site) TCCCCCGGGttatagaagctcgtactgtc

**His:Gpa-14**

PS95 F (*Xba*I site) CTAGTCTAGAgcgtttagttgttttgac

PS96 R (*Sma*I site) TCCCCCGGGttatgaaaggcccgttgc

**His:GPA-15**

PS113 F (*Xba*I) CTAGTCTAGAatgggatcaacgtgctcaacg

PS114 R (*Sma*I) TCCCCCGGGttattccatgcctgttcctcg

**His tag**

PS107 F (*Xho*I site) CCGCTCGAGatgcaccaccaccaccac

PS108 R (*Xho*I site) CCGCTCGAGaagcagccggatctctgtg

**GST pull-down constructs**

**gpa-14/ pET 24a**

PS43(*Sac*I) **CGAGCTCgcgtttagttgtttt**

PS40(*Hind*III) **CCCAAGCttttatgaaaggcccgt**

***dop-2c* /pIVEX2.3d**

PS59 F (*Nco*I) CATGCCATGGaggccggagag

PS80 R (*Sal*I) ACGCGTCGAC ttttttttttttttttttttttttttttttttagacatgcgcctg

Nucleotides added as overhangs to introduce restriction sites (indicated) are shown in uppercase and target sites used for amplifications are in lowercase.
